# Conservative Pediatric Duodenal Obstruction After Seatbelt Trauma: Why Monitoring Should Include Predefined Escalation Criteria, Not Gastric Tube Output Alone

**DOI:** 10.1002/ccr3.72909

**Published:** 2026-06-09

**Authors:** Muhammad Abdullah Awan

**Affiliations:** ^1^ Wah Medical College, National University of Medical Sciences Rawalpindi Pakistan

**Keywords:** conservative management, duodenal obstruction, nasogastric decompression, pediatric trauma, retroperitoneal hematoma, seatbelt injury

## Abstract

In pediatric duodenal obstruction caused by retroperitoneal hematoma, conservative management may be appropriate when stable; however, monitoring should include predefined escalation triggers combining nasogastric output, clinical status, inflammatory markers, nutrition, and selective imaging.

Dear Editor,

I read with great interest the case report by Matsumoto et al., describing conservative treatment of duodenal obstruction caused by retroperitoneal hematoma following seatbelt injury in a child. The report is clinically valuable because it highlights a rare pediatric trauma complication and shows that nonoperative management can succeed when hemodynamic stability is achieved and obstruction gradually resolves.

However, one practical aspect deserves further clarification. The authors state that gastric tube output was used as a key monitoring index to reduce repeated radiation exposure, which is reasonable in a pediatric patient. Nevertheless, gastric tube output alone may be insufficient as a decision‐making framework unless paired with predefined thresholds for escalation. In similar cases, clinicians need to know not only that conservative management is possible, but also when it should be abandoned.

This distinction is important because delayed escalation in traumatic duodenal obstruction could risk prolonged nutritional compromise, infected hematoma, worsening pancreatobiliary complications, intestinal ischemia, or missed perforation. A structured surveillance approach could include persistent high nasogastric output, progressive abdominal distension, fever, rising white blood cell count or C‐reactive protein, worsening pain, failure of contrast passage beyond the obstruction, hematoma enlargement, inability to advance enteral feeding, or evidence of cholangitis, pancreatitis, compartment physiology, or sepsis as triggers for repeat imaging, percutaneous drainage, endoscopic intervention, or surgery.

The authors appropriately emphasize the ALARA principle and the need to avoid unnecessary radiation exposure in children. Yet radiation‐sparing care should not be equated with imaging‐sparing care in all circumstances. Rather, selective imaging should be tied to clinical and biochemical deterioration, while bedside monitoring, serial examination, nutritional assessment, and laboratory trends should guide when imaging becomes justified. This would make conservative treatment more reproducible and safer for clinicians managing comparable pediatric trauma cases.

The case also raises an important nutritional point. Total parenteral nutrition was initiated when enteral feeding was not feasible, and oral intake was resumed after radiologic passage and decreasing gastric tube output. Future reports would be strengthened by specifying nutritional milestones, including when to start parenteral nutrition, when to reattempt enteral intake, and what output or clinical threshold supports oral advancement.

## 1 Conclusion

Matsumoto et al. [1] present an important example of successful conservative management of pediatric duodenal obstruction following seatbelt‐related retroperitoneal hematoma. To translate this experience into broader practice, future case reports and clinical protocols should define escalation criteria that combine nasogastric output with objective clinical, laboratory, nutritional, and imaging indicators. Such an approach would preserve the benefits of conservative, radiation‐conscious management while reducing the risk of delayed intervention.

## Author Contributions

**Muhammad Abdullah Awan:** conceptualization, data curation, formal analysis, project administration, writing – original draft, writing – review and editing.

## Funding

The author has nothing to report.

## Ethics Statement

The author has nothing to report.

## Consent

The author has nothing to report.

## Conflicts of Interest

The author declares no conflicts of interest.

## Data Availability

The author has nothing to report.
